# Bound nucleotide can control the dynamic architecture of monomeric actin

**DOI:** 10.1038/s41594-022-00743-5

**Published:** 2022-03-24

**Authors:** Rustam Ali, Jacob A. Zahm, Michael K. Rosen

**Affiliations:** 1 Department of Biophysics, Howard Hughes Medical Institute, UT Southwestern Medical Center, Dallas, TX USA; 2 Present Address: Department of Biological Chemistry and Molecular Pharmacology, Harvard Medical School, Boston, MA USA

**Keywords:** Thermodynamics, Actin

## Abstract

Polymerization of actin into cytoskeletal filaments is coupled to its bound adenine nucleotides. The mechanism by which nucleotide modulates actin functions has not been evident from analyses of ATP- and ADP-bound crystal structures of the actin monomer. We report that NMR chemical shift differences between the two forms are globally distributed. Furthermore, microsecond–millisecond motions are spread throughout the molecule in the ATP form, but largely confined to subdomains 1 and 2, and the nucleotide binding site in the ADP form. Through these motions, the ATP- and ADP-bound forms sample different high-energy conformations. A deafness-causing, fast-nucleating actin mutant populates the high-energy conformer of ATP-actin more than the wild-type protein, suggesting that this conformer may be on the pathway to nucleation. Together, the data suggest a model in which differential sampling of a nucleation-compatible form of the actin monomer may contribute to control of actin filament dynamics by nucleotide.

## Abstract (web summary)

NMR shows that ATP- and ADP-actin differ globally, including ground and excited state structures and dynamic architecture. Analyses of an actin mutant suggest the high-energy conformer of ATP-actin may be on the pathway to filament nucleation.

## Main

Dynamic rearrangements of the actin cytoskeleton are involved in many cellular processes, including motility, vesicle trafficking and division^1^. Disruption of these processes leads to numerous human pathologies^2,3^. In cells, actin cycles between monomeric globular (G) and filamentous (F) forms. Assembly of actin filaments occurs in two phases: (1) nucleation—the formation of an energetically unfavorable nucleus (dimer/trimer) from G-actin, followed by (2) elongation—rapid subunit addition to the nucleus and continued growth^4–7^. Bound adenine nucleotides control filament dynamics, with ATP favoring polymerization and ADP favoring depolymerization^8^.

**Fig. 1:**
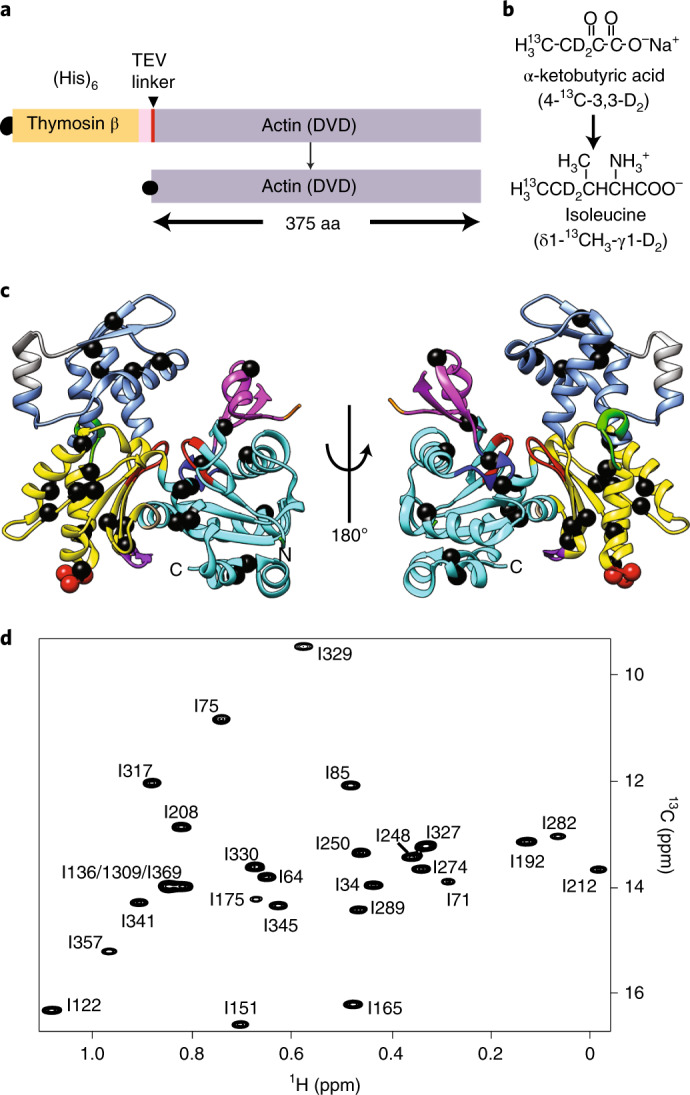
High-quality methyl TROSY NMR spectrum and sequence-specific isoleucine δ1-methyl chemical shift assignments of non-polymerizable (DVD) G-actin. **a**, Cartoon representation of TEV protease cleavable, (His)_6_-tagged DVD G-actin fused with thymosin β4. aa, amino acid. **b**, δ1 ^13^C-methyl labeling of isoleucine in *P. pastoris* using precursor α-ketobutyrate (4-^13^C-3,3-D_2_) (Methods). **c**, Ribbon diagram of G-actin (PDB 2HF4) showing isoleucine residues as black spheres. Elements of actin are colored as follows: SD1, cyan; SD2, magenta; SD3, yellow; SD4, corn blue; P1 and P2 nucleotide binding loops, red; sensor loop, blue; hydrophobic plug, green; WH2 binding motif in the hydrophobic groove, violet. Sites in SD3 mutated to block polymerization (D286A/V287A/D288A) are shown as red spheres. **d**, ^1^H/^13^C methyl TROSY NMR spectrum of perdeuterated, ^1^H/^13^C-Ile δ1-methyl-labeled G-actin showing assigned isoleucine residues (Extended Data Fig. 1).

To understand the physical mechanism underlying actin dynamics, the structures of actin monomers and filaments have been studied extensively using X-ray crystallography, electron microscopy (EM) and other tools. An important theme that has emerged from this work is that actin is a highly plastic molecule that can adopt many different conformations, often in nucleotide-dependent fashion. G-actin is composed of two lobes joined by a hinge at one end of the molecule (Fig. 1c). Each lobe is further divided into two subdomains (SDs), 1,2 and 3,4, linked through SDs 1 and 3. There is a deep cleft between SDs 2 and 4 (the nucleotide binding site (NBS)) that binds ATP or ADP and a divalent cation (Mg^2+^ in vivo). A shallower cleft at the base of the protein between SDs 1 and 3 (the hydrophobic groove) binds many actin ligands, including other actin molecules.

Structural studies of F-actin, initially by X-ray fiber diffraction^9,10^ and more recently by cryo-EM^11,12^ have advanced the mechanistic understanding of filament elongation, nucleotide hydrolysis and conformational changes in the protomers. Major findings of these studies are (1) that the conformation of G-actin in solution is quite different from the protomers in filament, with the latter being flatter due to rotation of the two lobes relative to each other^10^, (2) the existence of nucleotide-dependent closed and open states of protomers in F-actin^12^ and (3) opening of a hydrophobic pocket above the W-loop in the filament relative to the monomer^11^. Cryo-EM studies have visualized only internal segments of filaments. However, molecular dynamics simulations have provided models for changes in structures at the termini. In simulations, the terminal subunits of filaments tend toward conformations resembling G-actin. This behavior leads to opposite effects at the two filament ends, with different sets of unique contacts at each location, which produce different kinetics of monomer addition^13^.

In contrast to the growing structural understanding of filament behaviors, difficulties in measuring rare, transient oligomeric species have limited the understanding of the mechanism by which actin spontaneously nucleates—its detailed rate and equilibrium constants and the relevant structures. Recent modeling of actin assembly kinetics data has revealed that the low stability of the initially formed dimers and trimers is due to slow rates of monomer association to form these oligomers^14^. This analysis led to a mechanism in which monomers equilibrate in solution between a dominant nucleation-incompetent conformation and a weakly populated nucleation-competent conformation^14^. The existence of nucleation-competent activated intermediates in solution has also been suggested by previous experimental studies of actin, including proteolytic susceptibility^15^, fluorescence, UV and one-dimensional (1D) ^1^H NMR spectroscopies^16–20^ and thiol accessibility^21,22^. It remains unclear how the nucleotide state may modulate nucleation, as all studies we are aware of have studied the process only in the presence of ATP.

As for F-actin, a variety of data indicate that G-actin can adopt multiple conformations, some governed by nucleotide. This property is evident from several atomic-resolution structures of G-actin, which show alternate conformations of functionally important elements^1,23–25^, as well as radiolysis coupled with MS^26^. Mutagenic, biochemical and spectroscopic data suggest that conformational changes in actin arise through long-range, nucleotide-dependent, allosteric communication between the NBS and distant regions of the molecule, including the hydrophobic groove^27,28^, the sensor loop connecting the NBS to SD2^29^, and perhaps the D loop at the tip of SD2^30–32^. This communication controls affinity for proteins such as WH2 domains and cofilin, nucleotide exchange and filament elongation^33,34^. The presence of nucleotide-dependent allostery suggests that the actin monomer may be dynamic in solution, and that its dynamics may differ in the ATP and ADP forms, a feature observed in molecular dynamics simulations as well^35^. However, the dynamic fluctuations of G-actin in solution have not been directly examined, to our knowledge.

In this Article we report analyses of G-actin by NMR spectroscopy. Using methyl TROSY experiments in combination with mutagenesis, we have obtained sequence-specific chemical shift assignments of the δ1-methyl groups of all isoleucine residues (except overlapping I369/I136/I309) in G-actin, and used these to quantify microsecond–millisecond fluctuations across the protein in residue-specific fashion using relaxation dispersion measurements. Chemical shift analyses show that differences between the ground-state conformations of the ATP- and ADP-forms are widely distributed in the structure. Relaxation data suggest that, in the ATP-bound form, actin dynamics are concerted among the four SDs, whereas in the ADP-bound form they are confined mainly to the nucleotide binding cleft and SDs 1 and 2. Analysis of the excited-state chemical shifts revealed that the ATP and ADP forms each populate a distinct excited-state conformation. The microsecond–millisecond dynamics of an ATP-bound disease-causing, fast-nucleating actin mutant revealed an increased population of the excited state, suggesting that this state may be on the pathway to nucleation. Thus, dynamics of the actin monomer are controlled by the nucleotide switch, and might contribute to assembly of actin filaments through modulation of the rate of nucleation.

## Results

### Sequence-specific isoleucine δ1-methyl resonance assignments

To isotopically label actin for NMR analyses we expressed a non-polymerizable *Drosophila* 5C actin mutant, DVD (D286A, V287A, D288A), fused N-terminally to His_6_-tagged thymosin β4, in the methylotrophic yeast, *Pichia pastoris* (Fig. 1a)^36,37^. After affinity purification, the thymosin β4 was cleaved by specific proteolysis using tobacco etch virus (TEV) protease, leaving two additional amino acids at the N terminus of the protein. We enriched G-actin with ^13^C at the δ1-methyl position of isoleucine residues and highly deuterated all other aliphatic sites by growing yeast in minimal D_2_O medium supplemented with α-ketobutyric acid (methyl-^13^C, 99%; 3,3-D_2_, 98%) as previously described^37,38^ (Fig. 1b). To slow ATP hydrolysis and minimize actin self-assembly at the high concentrations and long times necessary for NMR experiments, we loaded the protein with Ca^2+^, which impedes both of these processes.

**Extended Data Fig. 1:**
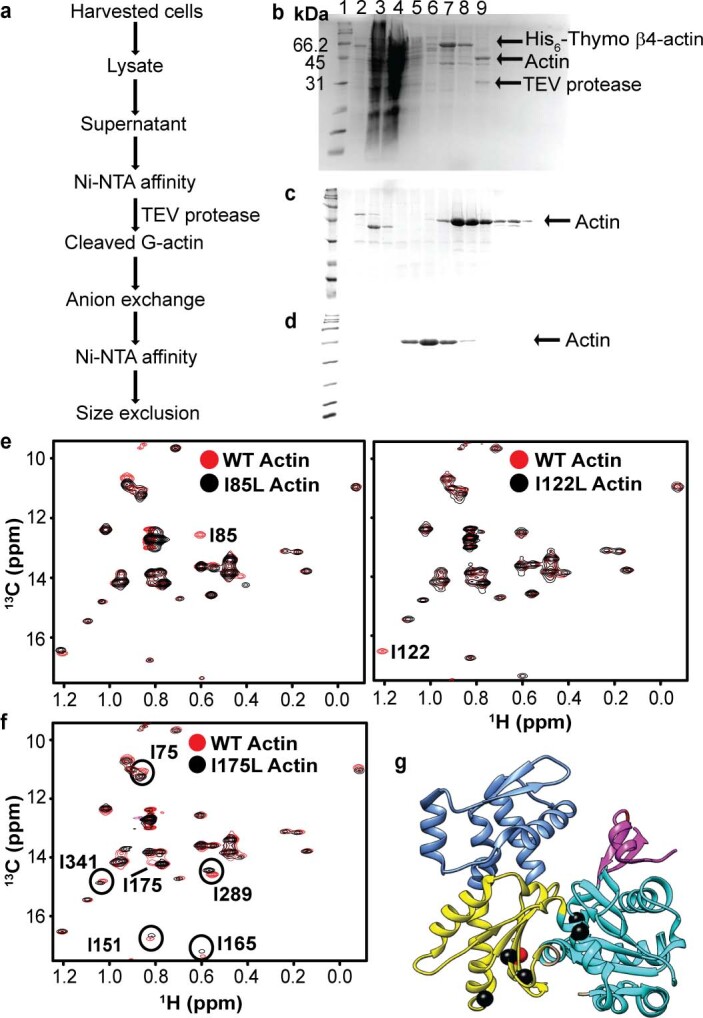
Expression, purification and δ1-methyl ^13^C assignments of G-actin. **a**, Schematics showing steps used for purification of recombinant G-actin from *P. pastoris*. **b-d**, SDS-PAGE (15%) showing fractions after Ni-NTA (**b)** anion exchange **(c)** and Superdex-200 size-exclusion chromatography **(d)**. In **(b)**, Lane 1, Molecular weight markers; Lane 2, pellet after lysis and centrifugation; Lane 3, supernatant after lysis and centrifugation; lane 4, flow through; lane 5, wash I; lane 6, wash II; lanes 7&8, elution; lane 9, after overnight TEV cleavage reaction. The SDS-PAGE gels are representative of at least 10 independent experiments. **e,f**, Overlaid ^1^H/^13^C HMQC spectra of WT G-actin **(red)** and mutants **(black)** I85L & I122L **(e)** and I175L **(f)**. All assignments were obtained by mutagenesis; missing peaks in the mutant spectra were used to obtain sequence specific assignments. The missing peak in each mutant spectrum is labeled. Overlaid spectra of WT and mutant I175L showing chemical shift perturbation experienced by residues that are away from the position of mutation and propagate to different sub-domains **(f)**. **g**, Ribbon diagram of G-actin with I175 shown as a red ball and residues altered by its mutation shown as black balls. Uncropped images for **b-d** are available as source data. Source data

There are 27 isoleucine residues in *Drosophila* 5C actin, distributed throughout the molecule in all four SDs (Fig. 1c). Figure 1d shows a 2D ^1^H/^13^C methyl TROSY spectrum of ^13^C Ile δ1-methyl-labeled DVD G-actin, in which all 27 expected resonances can be observed. All resonances except those corresponding to I309/I369/I136 (which are nearly overlapped) were assigned to individual residues using mutagenesis of Ile to Leu or Val (Fig. 1d and Extended Data Fig. 1e,f). Notably, during the course of assignment, we also observed that mutating certain residues caused chemical shift perturbations at other distantly located isoleucine sites (Extended Data Fig. 1f,g). For example, we observed chemical shift changes of residues I75, I341 (SD1) and I151, I165, I289 (SD3) when I175 in SD3 was mutated to V or L (Extended Data Fig. 1f,g). These effects suggest allosteric communication within the molecule.

### Nucleotide-dependent changes propagate throughout G-actin

**Fig. 2:**
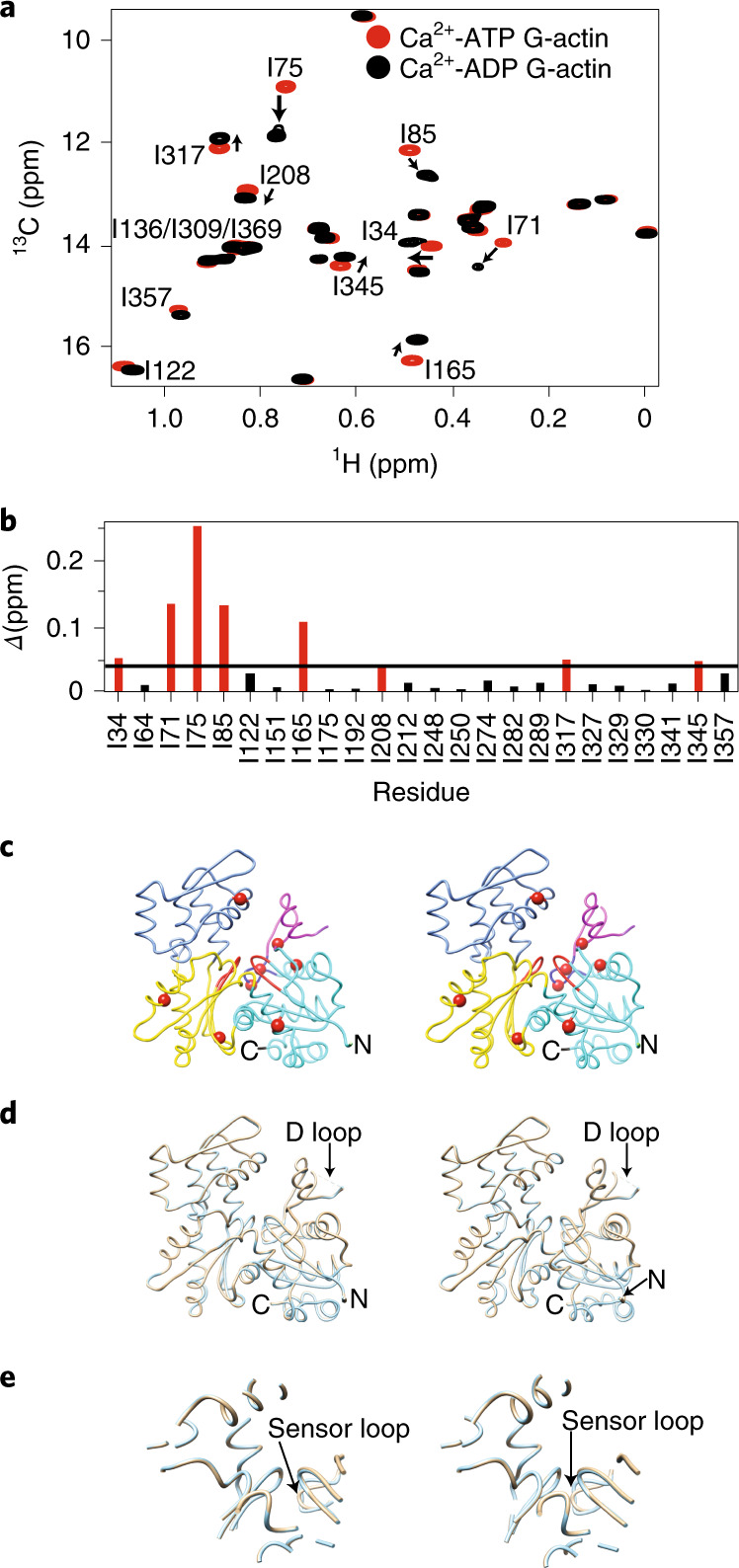
Chemical shift changes due to the nucleotide switch propagate throughout G-actin. **a**, Overlaid ^1^H/^13^C methyl TROSY NMR spectra of Ca^2+^-actin in the ATP- (red) and ADP-bound (black) forms. **b**,**c**, Chemical shift differences (*Δ* = [(δ^1^Η)^2^ + (0.25 × δ^13^C)^2^]^1/2^) between the ATP- and ADP-bound forms of actin. Residues showing *Δ* > 0.04 ppm (black line) are colored red (**b**), and are labeled in **a** and shown as red spheres in the wire representation of G-actin (PDB 2HF4) (**c**). SDs are colored as in Fig. 1c. **d**,**e**, Comparison of AP mutant G-actin crystal structures in the ATP- (PDB 2HF4; cyan) and ADP-bound (PDB 2HF3; wheat) forms^32^ (**d**). The structures in the two states are nearly identical except in the sensor loop region with an overall backbone r.m.s.d. of 0.23 Å (**e**). Panels **c**–**e** show stereo views.

**Extended Data Fig. 2:**
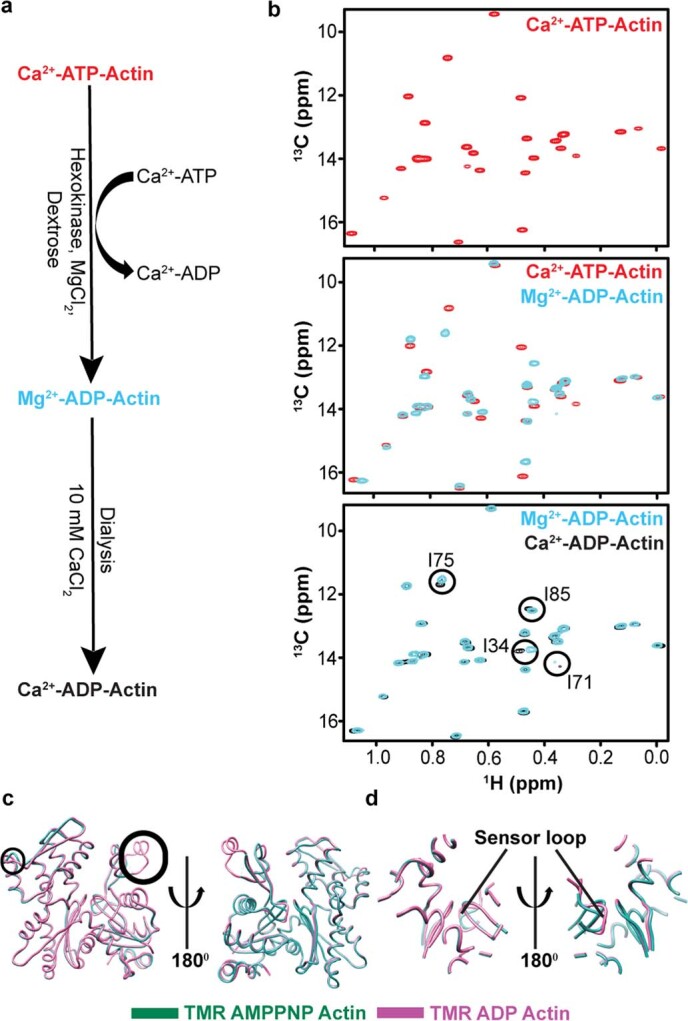
Preparation of Ca^2+^-ADP-actin and effects of metal ion on ^1^H/^13^C HMQC spectra. **a**, Purified Ca^2+^-ATP-actin was first converted using hexokinase to Mg^2+^-ADP-actin, which was then dialyzed against buffer containing Ca^2+^-ADP to produce Ca^2+^-ADP-actin. See Methods for details. **b**, ^1^H/^13^C HMQC spectra of perdeuterated, ^1^H/^13^C- Ile δ1-methyl-labeled Ca^2+^-ATP-actin **(top)**; overlaid spectra of Ca^2+^-ATP-actin **(red)** and Mg^2+^-ADP-actin **(cyan) (middle)**; overlaid spectra of Mg^2+^-ADP-actin **(cyan)** and Ca^2+^-ADP-actin **(black) (bottom)**. Residues showing chemical shift changes upon metal switching in the ADP state are circled. **c,d**, Structural comparison between AMPPNP (PDB ID INWK)- and ADP-bound (PDB ID 1J6Z) forms of TMR-labeled actin.

To understand how nucleotide affects the structure and dynamics of G-actin, we generated stable ATP and ADP forms bound to Ca^2+^ (Methods and Extended Data Fig. 2). Comparison of ^1^H/^13^C methyl TROSY spectra of these proteins revealed chemical shift differences for many resonances (Fig. 2a). Eight residues showed more than the average chemical shift difference between the two nucleotide forms (Fig. 2b). These residues are located in SD1 (I71, I75, I85, I345), SD2 (I34), SD3 (I165, I317) and SD4 (I208) of the G-actin structure (Fig. 2c). Mapping these residues onto the crystal structure of *Drosophila* actin, rendered non-polymerizable by A204E and P243K (AP) mutations, reveals that, in solution, the nucleotide switch propagates chemical shift changes and thus conformational and/or electrostatic changes across the protein to regions far from the NBS. By contrast, in crystals, the ATP- and ADP-bound forms of AP actin are nearly identical, with a backbone root-mean-square deviation (r.m.s.d.) of 0.23 Å (Fig. 2d,e), and all changes are restricted to elements within and surrounding the NBS, including the sensor loop^32^. Similarity is also seen in crystal structures of ATP- and ADP-bound tetramethylrhodamine-labeled actin (TMR-actin) (Extended Data Fig. 2c,d). Thus, in solution, the effects of the nucleotide switch are distributed more broadly across the actin molecule than appears to be the case in crystal structures.

We also observed chemical shift perturbations upon changing the bound divalent metal ion. Comparing the ^1^H/^13^C methyl TROSY spectra of Ca^2+^-ADP and Mg^2+^-ADP-actin revealed that four residues—I34, I71, I75 and I85—showed chemical shift differences between these states (Extended Data Fig. 2b, bottom). Unlike the differences due to nucleotide, these metal-induced changes are modest and restricted to the NBS and sensor loop.

### Pervasive microsecond–millisecond dynamics in ATP-bound G-actin

**Extended Data Fig. 3:**
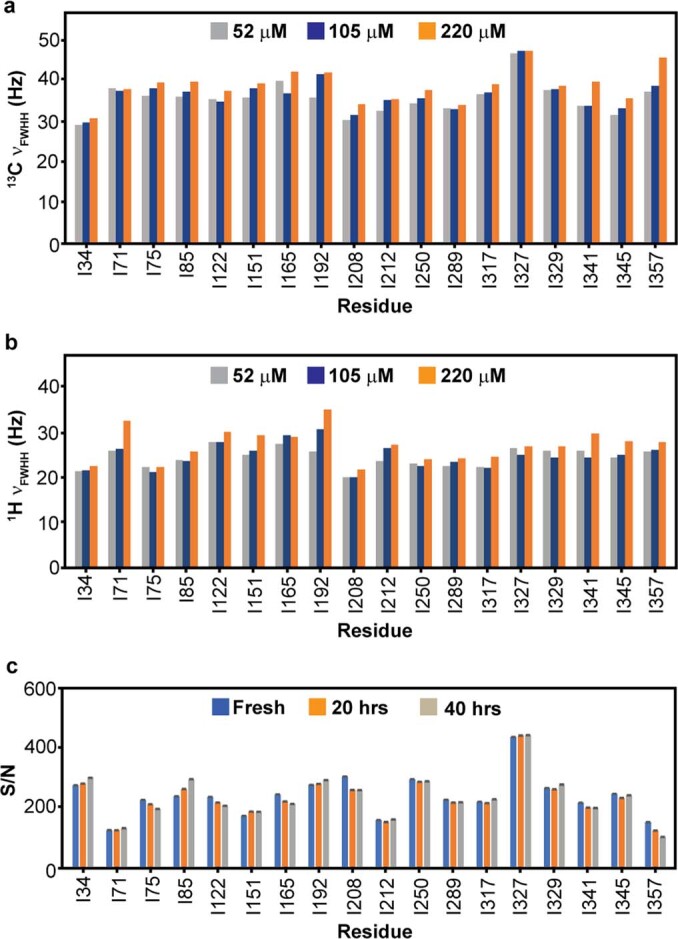
G-actin does not aggregate and is stable during NMR data acquisition at 100 μM. **a,b**, Full linewidth at half height (ν_FWHH_) in the ^13^C **(a)** and ^1^H **(b)** dimensions of resolved Ile δ1-methyl resonances in ^1^H/^13^C HMQC spectra recorded at 600 MHz on ^1^H/^13^C- Ile δ1-methyl-labeled Ca^2+^-ATP-actin at the indicated protein concentrations. **c**, S/N (signal to noise) ratio in ^1^H/^13^C HMQC spectra of ^1^H/^13^C- Ile δ1-methyl-labeled labeled Ca^2+^-ATP-actin acquired at different times (0, 20 and 40 hours) after sample preparation. No significant changes in S/N were observed over the 40 hour timecourse. Error bars represent SD calculated from the noise in the spectra.

We sought to characterize the microsecond–millisecond internal dynamics in G-actin by NMR relaxation dispersion measurements. To ensure that these dynamics reflect internal motions of the protein rather than transient intermolecular contacts, we first acquired ^1^H/^13^C heteronuclear multiple quantum coherence (HMQC) data at different protein concentrations and compared methyl ^1^H and ^13^C linewidths for each resolved peak. An increase in linewidth would be an indication of self-association, which would complicate interpretation of the relaxation data. We found that linewidths were generally similar up to a concentration of ~100 µM but increased slightly at 220 µM concentration (Extended Data Fig. 3a,b), indicating that G-actin does not self-associate appreciably at the lower concentrations. Thus, we acquired all Carr–Purcell–Meiboom–Gill (CPMG) data at G-actin concentrations of 100 ± 10 μM. Similarly, to ensure that the sample quality did not change during the course of NMR data acquisition, we measured the signal-to-noise ratio of all resonances at different time intervals in ^1^H/^13^C methyl TROSY spectra (Extended Data Fig. 3c). This analysis showed that, within experimental error, there were no changes to the spectra after 40 h, except for I357 at the C terminus of the protein, suggesting some degradation of the disordered G-actin tail but no larger-scale changes to the molecule (Extended Data Fig. 3c).

**Fig. 3:**
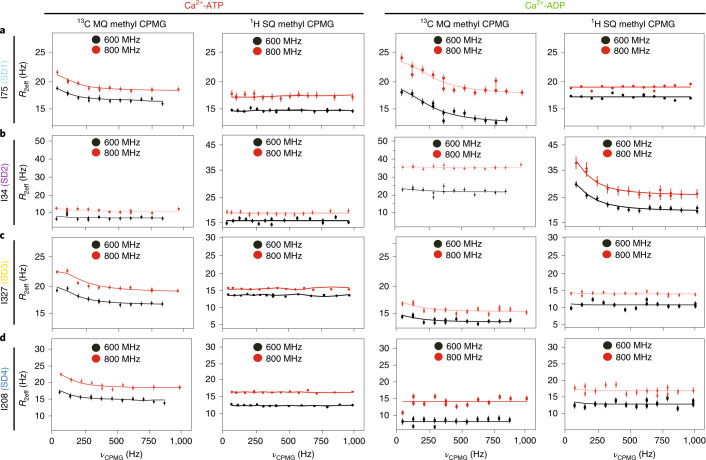
Microsecond–millisecond dynamics measurements of Ca^2+^-ATP and -ADP-actin. **a**–**d**, Representative relaxation dispersion profiles (effective relaxation rate *R*_2eff_ versus CPMG frequency) for isoleucine residues in SD1 (**a**), SD2 (**b**), SD3 (**c**) and SD4 (**d**). In all panels, data recorded at 600-MHz and 800-MHz field strengths are shown in black and red, respectively. MQ data (columns 1 and 3) report on the chemical exchange experienced by both ^1^H and ^13^C nuclei, whereas SQ data (column 2 and 4) report on the chemical exchange experienced by ^1^H only. Error bars represent s.d., calculated from the two and three replicated data points for 600- and 800-MHz data, respectively.

**Extended Data Fig. 4:**
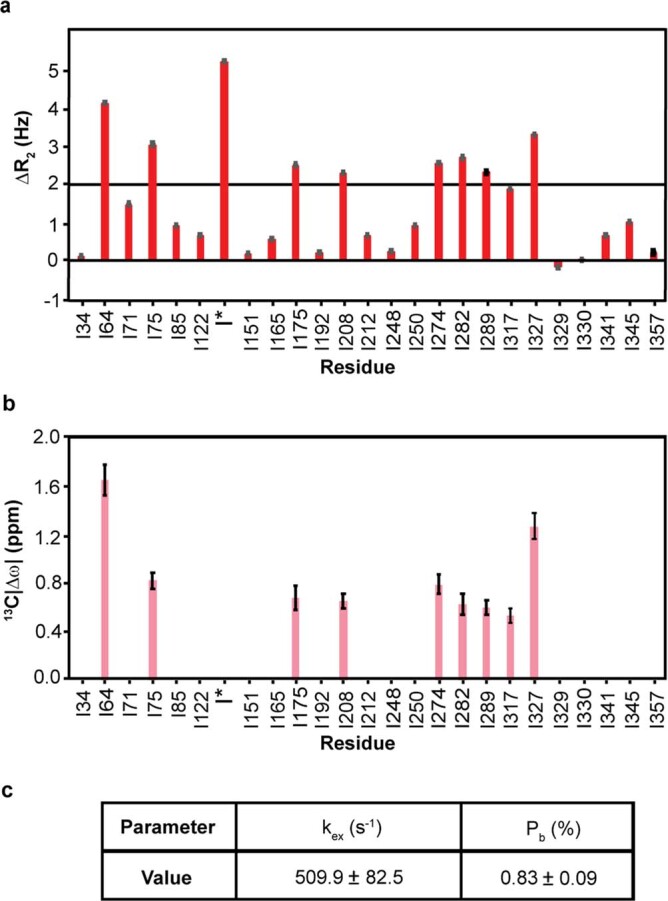
μs-ms dynamics of Ca^2+^-ATP-actin. **a**, Multiple quantum relaxation dispersion (ΔR_2_), the difference in R_2eff_ at lowest and highest pulsing rates, recorded at 800 MHz, of Ile δ1-methyl resonances of ^1^H/^13^C- Ile δ1-methyl-labeled Ca^2+^-ATP-actin. Error bars in ΔR_2_ represent the SD based on noise levels in NMR spectra and were calculated using standard error propagation methods**, b**, Magnitude of ^13^C chemical shift difference (^13^C|Δω|) between the major (ground) and minor (excited) states for Ile δ1-methyl groups of ATP-actin. **c**, Thermodynamic (P_b_) and kinetic parameters (k_ex_) obtained from two-state global fitting of the relaxation dispersion data. Errors in ^13^C|Δω|, P_b_, and k_ex_ were determined from the covariance matrix of the global fits. ΔR_2_ values near zero were obtained for all Ile δ1-methyl resonances in a ^1^H single quantum relaxation dispersion experiment as shown for representative residues in Fig. 3**(columns 2 and 4)**.

Given the sample concentration limits and to exploit the methyl TROSY effect, we acquired ^1^H/^13^C multiple quantum (MQ) CPMG relaxation dispersion data^39,40^ on fully deuterated ^1^H/^13^C Ile δ1-methyl-labeled DVD G-actin bound to ATP. In these experiments, methyl sites undergoing conformational exchange on the microsecond–millisecond timescale show changes in their effective relaxation rate (*R*_2eff_) with the frequency of refocusing pulses (*ν*_CPMG_). When dynamics are modeled as the interconversion between two states, *R*_2eff_ is a function of *k*_ex_, the sum of the forward and reverse rate constants, the populations of the minor (excited) state and the absolute value of chemical shift difference between the major (ground) and minor (excited) states. The chemical shift changes for a given nucleus reflect differences in its electronic environment between the two states. The first column of Fig. 3 shows ^1^H/^13^C MQ CPMG relaxation dispersion profiles for representative residues in each SD of Ca^2+^-ATP-actin. A total of nine resolved methyl resonances (I64, I75, I175, I208, I274, I282, I289, I317 and I327) out of 27 showed a relaxation dispersion (the difference in *R*_2eff_ at fast and slow pulsing limits) >2 Hz (Extended Data Fig. 4a). These correspond to residues spread over all SDs of the molecule, indicating pervasive motion on the microsecond–millisecond timescale. We also observed significant relaxation dispersion for one or more of the overlapped resonances corresponding to I136/I309/I369. To extract the kinetic and thermodynamic parameters of these motions, as well as the ^1^H and ^13^C chemical shifts of the relevant states, we acquired both ^1^H/^13^C MQ CPMG data and TROSY-based ^1^H single-quantum (SQ) CPMG^41^ data at 600- and 800-MHz field strengths (second column, Fig. 3). Comparison of ^1^H SQ and ^1^H/^13^C MQ relaxation dispersion profiles distinguishes the contributions to relaxation of ^1^H and ^13^C nuclei. The small relaxation dispersion in the ^1^H SQ CPMG profiles (generally <1 Hz) indicates that proton nuclei do not make significant contributions to the MQ relaxation dispersion, indicating the structural transitions between the ground and excited states cause changes primarily in ^13^C chemical shifts. Simultaneous fitting of the SQ and MQ relaxation dispersion data acquired at 600 and 800 MHz according to a two-state model of chemical exchange^40,42^ yielded an exchange rate of 510 ± 80 s^−1^ and a minor-state population of 0.83 ± 0.09% (Extended Data Fig. 4b,c), as well as the ^1^H/^13^C chemical shift differences between ground and excited states. The fact that the relaxation data of all resonances could be fit simultaneously with the reduced *χ*^2^ value (*χ*^2^/(degrees of freedom)) converged to ~1 indicates that the data are consistent with a single dominant dynamic process. Furthermore, because the resonances involved represent methyl groups distributed across the protein, these dynamics span the structure.

### Dynamics in ADP-actin are largely restricted to SDs 1 and 2

**Extended Data Fig. 5:**
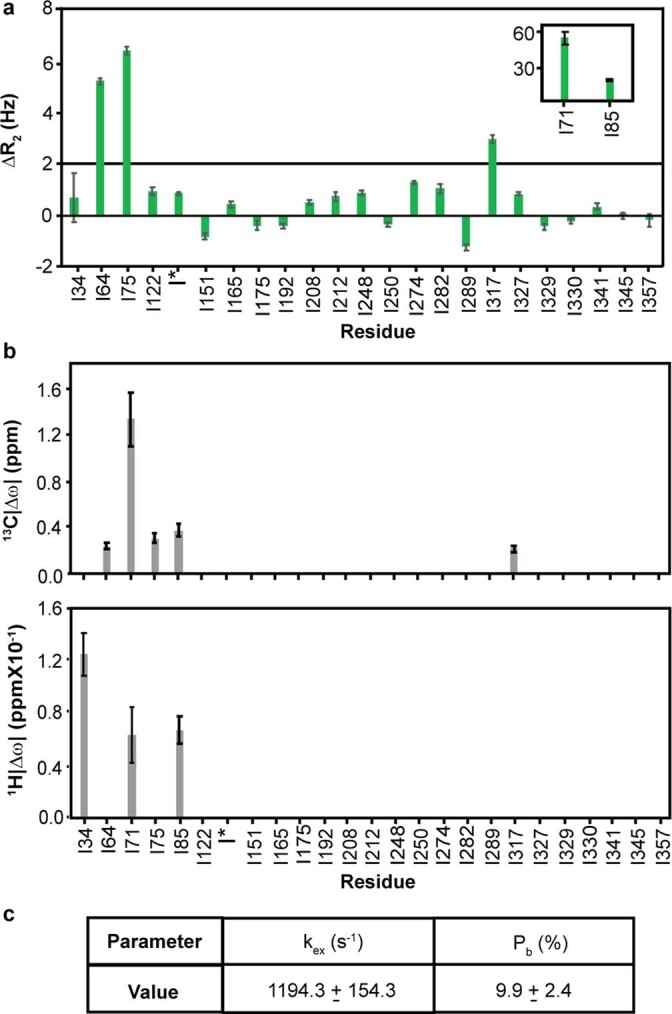
μs-ms dynamics in Ca^2+^-ADP-actin. **a**, Multiple quantum relaxation dispersion (ΔR_2_), difference in ^1^H/^13^C R_2eff_ between the lowest and highest CPMG field strengths, recorded at 800 MHz of Ile δ1-methyl resonances of ^1^H/^13^C- Ile δ1-methyl-labeled Ca^2+^-ADP-actin. Error bars in ΔR_2_ represent the SD based on noise levels in NMR spectra and were calculated using standard error propagation methods **b**, Absolute value of the difference between ground and excited state ^13^C and ^1^H chemical shifts |Δω| for Ile δ1-methyl resonances of Ca^2+^-ADP-actin determined from global fitting of the MQ and ^1^H SQ CPMG data. **c**, Thermodynamic (P_b_) and kinetic parameters (k_ex_) obtained from two-state global fitting. Errors in |Δω|, P_b_, and k_ex_ were determined from the covariance matrix of the global fits.

Figure 3 shows ^1^H/^13^C multiple (column 3) and ^1^H single (column 4) quantum relaxation dispersion profiles for four residues in Ca^+2^-ADP-actin. A total of six resolved methyl resonances (I34, I64, I71, I75, I85 and I317) of 27 showed substantial relaxation dispersion (>2.0 Hz) in either ^1^H/^13^C MQ or ^1^H SQ datasets recorded at 600- and 800-MHz field strengths (Extended Data Fig. 5). All of these residues except I64 are also perturbed by changing nucleotide state (Fig. 2). Unlike for ATP-actin, the ^1^H/^13^C MQ relaxation dispersion for ADP-actin has substantial contributions from ^1^H relaxation for several resonances (I34, I85 and I71; Fig. 3, fourth column and Extended Data Fig. 5b). This indicates that the structural transitions cause changes to both ^1^H and ^13^C chemical shifts of the ADP-bound protein. As with ATP-actin, simultaneous fitting of the ^1^H/^13^C MQ and ^1^H SQ data acquired at 600- and 800-MHz field strengths in ADP-actin to a two-state chemical exchange model yielded an exchange rate of 1,194 ± 154 s^−1^ and a minor-state population of 10 ± 2.4% (Fig. 5c). Thus, in its ADP form, actin samples an excited state to a 12-fold greater degree than it does in its ATP form. Moreover, except for I317, the residues showing dynamics are restricted to SDs 1 and 2 of the protein.

### NBS hydrogen bonding may couple the two lobes of actin

**Fig. 4:**
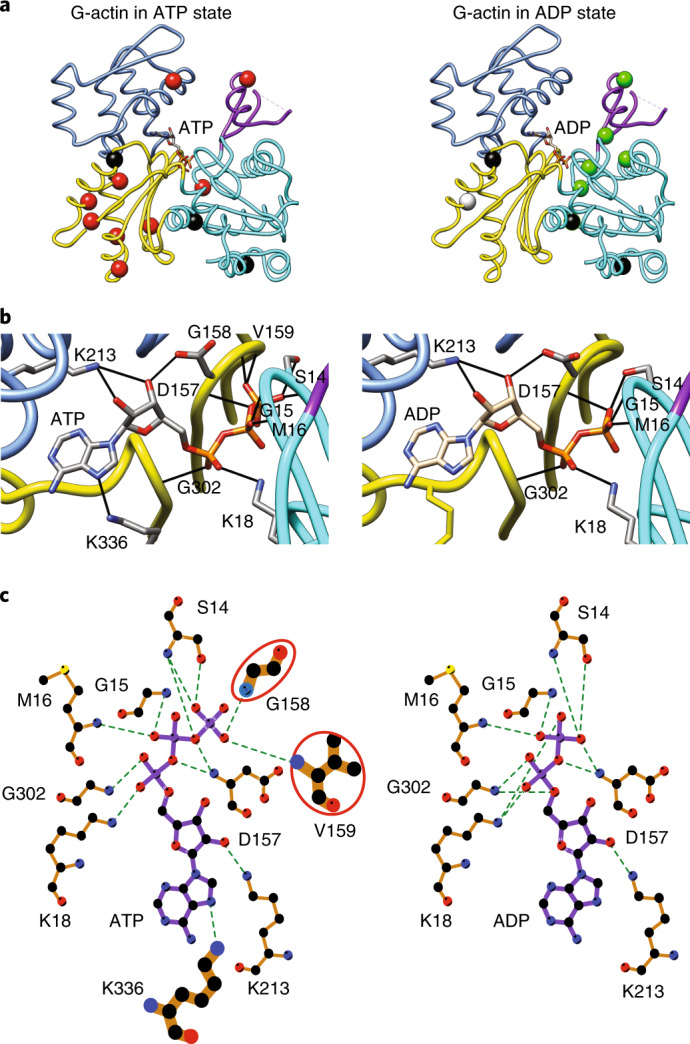
G-actin has a different dynamic architecture in its ATP- and ADP-bound forms. **a**–**c**, Isoleucine residues with MQ or ^1^H SQ relaxation dispersion > 2 Hz mapped onto the G-actin structure in the ATP- (left panel, red balls; PDB 2HF4) and ADP-bound (right panel, green balls; PDB 2HF3) forms (**a**). Black balls, overlapped residues (I136, I309, I369) in NMR spectra. The SDs are colored as in Fig. 1c. Panels **a** and **b** were generated using Chimera. 3D (**b**) and 2D (**c**) depictions of the NBS of ATP- and ADP-bound actin (left and right images, respectively). Hydrogen bonds are indicated by dashed lines. In ATP-actin, the two halves of the structure are bridged through additional hydrogen bonds between the terminal γ-phosphate and residues in SD3 that are absent in ADP-actin (circled in red). Panel **c** was generated using LigPlot^+^.

**Extended Data Fig. 6:**
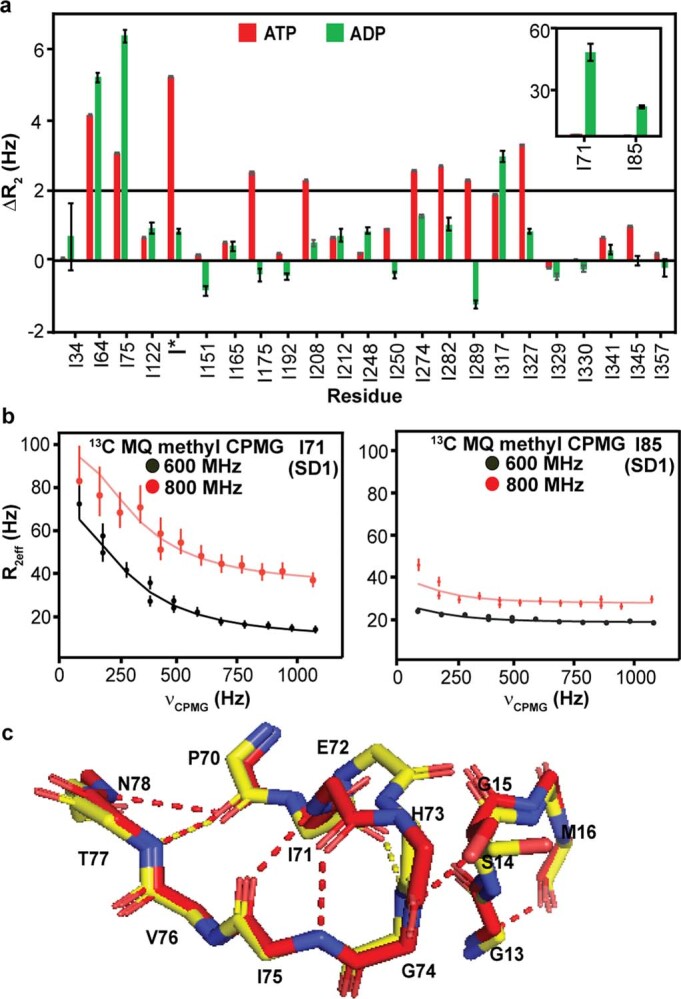
Comparison of dynamics in ATP- and ADP-bound G-actin. **a**, Comparison of MQ ΔR_2_ recorded at 800 MHz of Ile δ1-methyl resonances of ^1^H/^13^C- Ile δ1-methyl-labeled ATP- **(red)** and ADP-bound **(green)** actin. Only residues I64, I71, I75 and I85 showed ΔR_2_ ≥ 2 Hz in ADP-actin, whereas many additional residues showed ΔR_2_ ≥ 2 Hz in ATP-actin. Error bars in ΔR_2_ represent the SD, based on noise levels in NMR spectra and were calculated using standard error propagation methods **b**, Although, residues showing ΔR_2_ ≥ 2 Hz are mainly confined to SD1 and SD2, in ADP-actin, the magnitude of ΔR_2_ was higher due to a greater population of the excited state. The error bars represent the SD (standard deviation), calculated from the two and three replicated data points for 600 and 800 MHz data, respectively.**c**, Hydrolysis of ATP **(red)** into ADP **(yellow)** triggers a series of rearrangements from which multiple hydrogen bonds (shown as dotted line) are broken.

We next compared the dynamics of ATP- and ADP-actin considering the hydrogen-bond connectivity in the NBS. Figure 4 shows residues with either ^1^H/^13^C MQ or ^1^H SQ relaxation dispersion >2 Hz (Extended Data Fig. 6a), mapped onto the G-actin structure. In the ATP-bound form (Fig. 4a, left) the dynamics are distributed throughout all SDs of the molecule—SD1 (I75), SD2 (I64), SD3 (I175, I274, I282, I289, I317, I327) and SD4 (I208). Thus, the entire molecule appears to undergo collective motion. This motion provides a probable explanation for biochemical data indicating functional coupling between the NBS and the hydrophobic groove, the sensor loop in SD1 and the D loop in SD2. By contrast, except for I317 (SD3), residues showing dynamics in the ADP-bound form are all located in SD2 (I34, I64) or SD1 (I71, I75 and I85) (Fig. 4a, right). These observations suggest that, in contrast to the ATP-bound form, when bound to ADP the two lobes of actin are largely uncoupled (in the context of processes on the microsecond–millisecond timescale). Thus, the nucleotide switch controls the mechanical organization of the actin monomer.

Analysis of the hydrogen-bonding networks in the crystal structures of ATP- and ADP-actin (PDB 2HF4 and 2HF3, respectively) suggests a possible explanation for the differences in dynamics architecture of the two forms of the protein. Nucleotides in G-actin are bound to the P1 and P2 loops in SD1 and SD3, respectively. In the ATP form, the γ-phosphate of the nucleotide hydrogen-bonds to G158 and V159 in SD3, and also S14 in SD1 (Fig. 4b, left), connecting the two lobes of the protein near the sensor loop (which is hydrogen-bonded to the S14 side chain hydroxyl group). However, in the ADP form, the hydrogen bonds to G158/V159 are lost, and S14 moves to hydrogen-bond with the β-phosphate (Fig. 4b, right). These changes effectively shift the connection between the two lobes toward the center of the protein (forward in the orientation of Fig. 4a), away from the sensor loop. Furthermore, the movement of S14 disrupts hydrogen bonds of its hydroxyl group to the backbone amides of H73 and G74 in the sensor loop, breaking the contacts of this element to the P1 loop. In response, the sensor loop adopts a different average conformation and also becomes more dynamic (Extended Data Fig. 6b,c), populating an excited state to ~10%, versus ~1% in the ATP form. These data suggest that the hydrogen bonds from SD3 and SD1 to the γ-phosphate in ATP-actin mechanically couple the two lobes of the protein, causing them to fluctuate concertedly. In the presence of ADP, these interactions are lost, and the P1 loop and sensor loop relax, relieving tension between the two lobes. This mechanism would explain why dynamics in the ATP form are widespread throughout the actin molecule, whereas in the ADP form they are localized near the NBS in SD1 and SD2.

### Different excited-state conformations of ATP- and ADP-actin

**Fig. 5:**
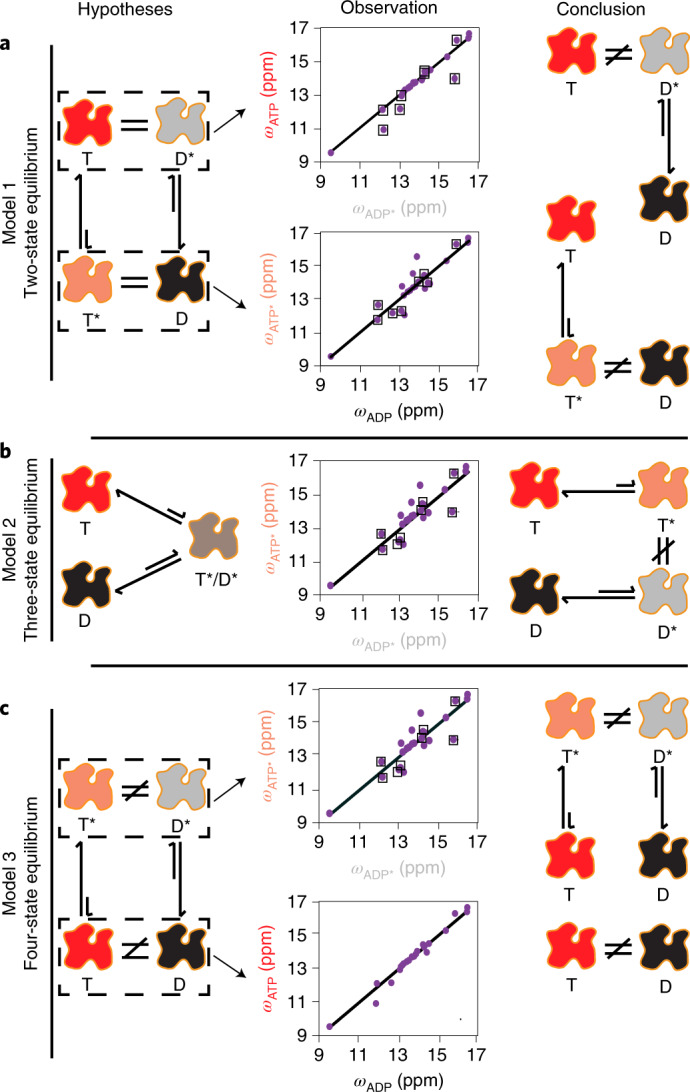
Structural models for the dynamics of G-actin. In each panel the left column schematically depicts a potential structural mechanism to explain the observed dynamics (T and D indicate ATP- and ADP-bound actin ground states, respectively, and T* and D* indicate the respective excited states), the middle panels plot various ground- and excited-state chemical shifts against each other, and the right column depicts the mechanistic conclusion from the data (see main text). In the middle column, each datapoint represents an isoleucine residue. Residues with different ground-state chemical shifts in ATP- and ADP-actin are boxed, and are not used to make structural comparisons. **a**, Model in which actin exists in only two conformational states, which are differentially biased by ATP and ADP (left). In such a model, T and D* are conformationally identical and have the same chemical shifts, as do D and T*. The scatter plots (middle) compare the ATP and ADP* ^13^C chemical shifts (top) and the ADP and ATP* chemical shifts (bottom). Off-diagonal, non-boxed points indicate structural differences between the states (more definitive for the T*/D comparison than the T/D* comparison), ruling out a two-state equilibrium (right). **b**, In a three-state model, ATP- and ADP-actin have distinct ground-state structures, but populate a common excited state (left). The scatter plot of ATP* versus ADP* ^13^C chemical shifts (middle) shows numerous off-diagonal, non-boxed points, indicating the two forms do not populate a common excited state (right). **c**, In a four-state equilibrium, ATP- and ADP-actin have distinct ground- and excited-state conformations (left). The scatter plots (middle) of ATP* versus ADP* (top) and ATP versus ADP (bottom) ^13^C chemical shifts show numerous off-diagonal, non-boxed points, indicating four distinct conformations (right).

We next examined the structural nature of the actin dynamics by comparing the excited- and ground-state chemical shifts in the ATP and ADP forms of the protein. We considered three possible models of the system. In the first, which has been proposed for a trimeric G protein (another nucleotide-dependent switch)^43^, actin can adopt only two interconverting conformations, and these are differentially favored by ATP and ADP. Thus, in this model, the excited state of the ATP form (ATP*) is the same as the ground state of the ADP form (ADP), and vice versa (Fig. 5a). The middle panels of Fig. 5a compare the chemical shifts of the ATP-actin ground state (ATP) and the ADP-actin excited state (ADP*) and vice versa. Excluding resonances that show chemical shift differences between the ATP- and ADP-form ground states (boxed), which may differ simply because of the chemical differences between the two nucleotides, the chemical shifts of ADP do not coincide with those of ATP*, ruling out a two-state model (Fig. 5a, right). Note that no conclusion can be drawn from the opposite comparison of ADP* with ATP, because all off-diagonal peaks, except I64, have chemical shift differences between the ATP- and ADP-form ground states. In the second model, which invokes three conformations, ATP- and ADP-actin have different ground states but sample a common excited state. In this three-state equilibrium (Fig. 5b, left), the ATP* and ADP* chemical shifts would be identical (again, except for resonances that respond simply to the chemical differences between the nucleotides). Yet the middle panel of Fig. 5b shows that the ATP* and ADP* chemical shifts are quite different, ruling out this model as well (Fig. 5b, right). Our data then lead to a simplest model where the conformations of ATP, ADP, ATP* and ADP* are all different (Figs. 2b and 5b) and interconvert in a four-state equilibrium (Fig. 5c). More complex possibilities with additional states could also be correct, but we cannot distinguish them with our data.

### A fast-nucleating mutant populates ATP* to a greater degree

**Fig. 6:**
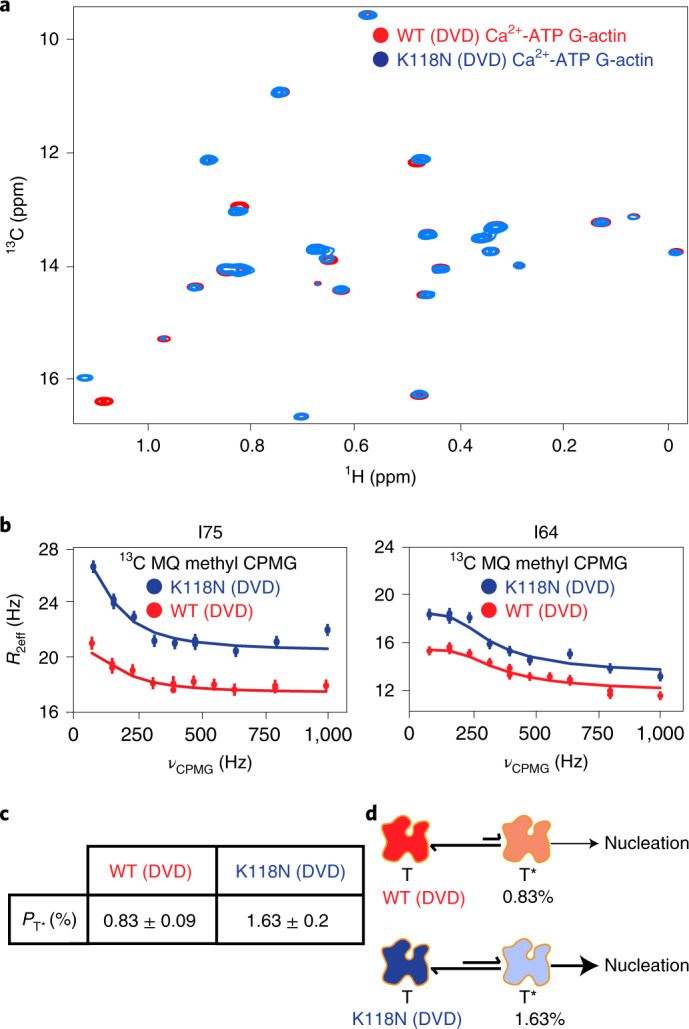
A disease-causing, fast polymerizing actin mutant, K118N, increases the population of the ATP* state. **a**, Overlaid ^1^H/^13^C methyl TROSY NMR spectra of ATP-bound wild-type (WT, red) and K118N G-actin (blue). **b**, MQ relaxation dispersion profiles for representative isoleucine resonances in WT (red) and K118N (blue) G-actin. The error in measurements was calculated from the intensities of duplicate data points (Methods). **c**, Comparison of ATP* populations in WT and K118N G-actin. **d**, Proposed model for the increased polymerization rate of the K118N mutant actin based on a higher population of an on-pathway intermediate in filament formation, T*. Errors in the population of excited states (*P*_T*_) were determined from the covariance matrix of the global fits.

**Extended Data Fig. 7:**
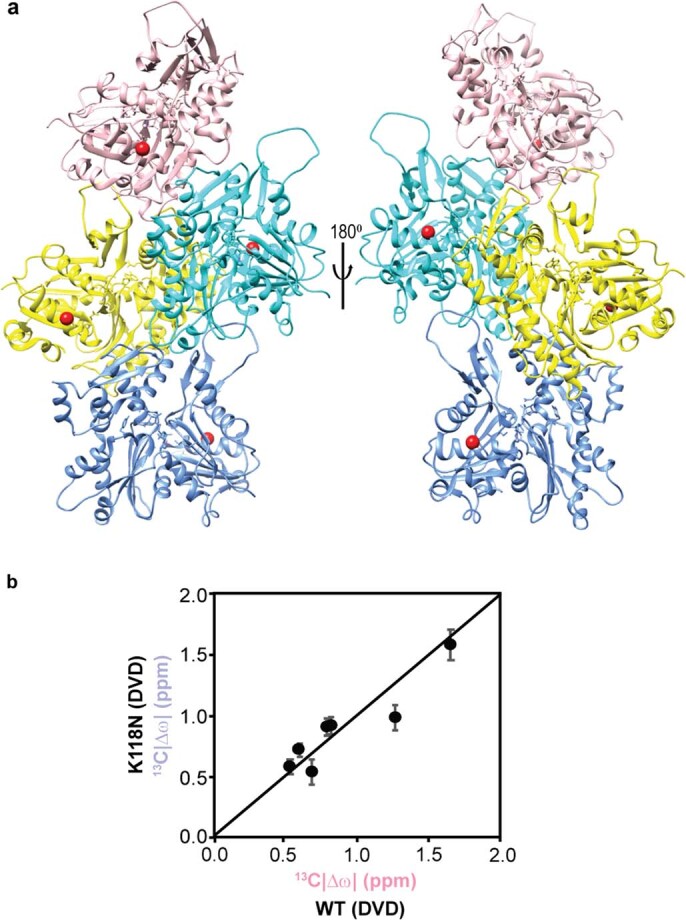
Structural and dynamic analyses of K118N Ca^2+^-ATP-actin. **a**, Location of K118 on the F-actin structure (PDB ID 6DJM). K118, shown as a red sphere, is not at a subunit interface. **b**, Comparison of Ile δ1-methyl ^13^C excited state chemical shifts ^13^C|Δω| of K118N **(light blue)** and WT **(pink)** ATP-actin. Error in ^13^C|Δω|, P_b_, and k_ex_ were determined from the covariance matrix of the global fits.

The highly conserved lysine 118 of human γ-actin, equivalent to lysine 118 in the *Drosophila* 5C actin here, is mutated to asparagine in autosomal-dominant, nonsyndromic, early-onset deafness^44^. This mutation increases the rate of actin nucleation, but is not located at any of the subunit interfaces in the actin filament, nor is it proximal to the NBS (Extended Data Fig. 7). To understand the mutant, and more generally to determine whether actin dynamics may be functionally important, we examined the ATP-bound K118N mutant of DVD *Drosophila* 5C actin. Figure 6a shows overlaid methyl TROSY spectra of the wild-type and K118N DVD actins. Except for residue I122, which is located in the same helix as K118, none of the isoleucine resonances showed significant chemical shift differences between the two proteins, indicating that the K118N mutation only minimally perturbs the ground-state structure. In ^1^H/^13^C MQ relaxation dispersion experiments, the K118N mutant showed an identical pattern of dynamic resides (with *R*_2eff_ > 2 Hz) as the K118 DVD protein. However, the magnitude of relaxation dispersion (*R*_2eff_) in the mutant was generally approximately two times higher than in the wild-type protein (Fig. 6b), suggesting an increased population of the excited state. Consistent with this idea, global fitting of ^13^C SQ and ^1^H/^13^C MQ relaxation dispersion data acquired at 600 and 800 MHz yielded a minor-state population of 1.6 ± 0.2% and *k*_ex_ of 450 ± 55 for the K118N mutant (Fig. 6c). The chemical shifts of the ATP* state for the K118 and K118N DVD proteins are nearly identical (Extended Data Fig. 7b), indicating that the two proteins sample the same excited-state conformation. Thus, the NMR data indicate that the K118 and K118N DVD proteins sample the same conformational equilibrium, but the latter populates the excited state to an approximately twofold greater degree. The parallel increases in excited-state population and actin nucleation rate suggest that the two processes may be related. Taken together, NMR and biochemical data thus lead to a model in which the ATP* state of actin is ‘on pathway’ to nucleation (Fig. 6d), consistent with a recent kinetic model for this process (see below). The K118N mutant, because it populates this state more than the wild-type protein, nucleates more rapidly. This model generally posits that the microsecond–millisecond dynamics of ATP-actin are functionally important because they sample a conformation that promotes nucleation of filaments.

## Discussion

The nearly identical crystal structures of ATP- and ADP-actin have made it difficult to understand how nucleotide controls many aspects of actin biochemistry. Here we have shown, by NMR, that differences between the two nucleotide forms are widespread in the protein, including both the ground-state structure and dynamic architecture. Correlations between dynamics and nucleation suggest that motions of the actin monomer may contribute to nucleotide-dependent assembly behavior. As a caveat, we note that, because we were unable to examine ATP-actin in the presence of Mg^2+^, it is possible that some of the chemical-shift and dynamic properties we observe, and our conclusions from them, are specific to the Ca^2+^-bound state.

Our NMR data indicate that structural differences between the ground states of ATP- and ADP-actin are global in nature and not confined to the nucleotide binding elements. This is consistent with indirect observations of conformational changes in solution^45–47^ and molecular dynamics simulations suggesting that the two nucleotide forms adopt different degrees of twist between the two lobes^35^. Sensor loop residues I71 and I75, which are adjacent to the NBS, showed the largest chemical shift differences between the two nucleotide forms, consistent with conformational changes in this region of both the TMR-labeled and AP actin crystal structures^30–32^. Additionally, however, residues I345 and I85 (SD1), I34 (SD2), I317 (SD3) and I208 (SD4), which are between 12 Å and >20 Å away from the NBS, also showed significant chemical-shift differences, indicating that conformational changes propagate to distant regions. The W-loop (residues 165–172), which lines the hydrophobic groove, contacts numerous actin ligands, including WH2 domain proteins and longitudinally adjacent monomers in the filament. Isoleucine 165 has among the largest chemical shift differences between the ATP- and ADP-forms, indicating that nucleotide-dependent conformational changes propagate to the hydrophobic groove, probably accounting for nucleotide-dependent affinities of most ligands that contact this region^48–53^. It is possible that changes in this region reflect opening of the W-loop, which is necessary for longitudinal contacts in the actin filament (see Discussion)^14^. Thus, our chemical-shift data suggest the presence of allosteric networks that cause global changes in the ground state of actin with changes in nucleotide.

ATP- and ADP-actin also show differences in dynamic behavior. Both forms of the protein have appreciable dynamics on the microsecond–millisecond timescale. In ATP-actin, residues with measurable dynamics are located (1) at the SD interfaces (I75 and I85 at SD1–SD2, I175 near SD1–SD3, I274 at SD3–SD4), (2) in regions that make intersubunit contacts in the actin filament (I64 in the SD2 and at the end of the DNase I loop, I289 and I208 at the tips of SD3 and SD4, respectively) and (3) near the catalytic site (I175). Thus, dynamics propagate to all SDs, suggesting a network of allosteric interactions that span the entire protein. The relaxation data for all methyl groups in ATP-actin can be fit to a model of a single dynamic process, consistent with concerted motion in which all of the SDs are coupled together. The presence of dynamics in regions that make intersubunit contacts in actin filaments and or near the catalytic site suggest that the motions might contribute to filament assembly (see below) and/or ATP hydrolysis. In contrast to the ATP-bound form, in the ADP complex, residues showing dynamics are located in SD1 (I71, I75 and I85) and SD2 (I34 and I64), indicating a dynamic network that is broken at the SD1–SD3 interface, restricting motion to SD1 and SD2. The excited-state chemical shifts show that actin samples different high-energy conformations in the two nucleotide forms. Collectively, our data show that actin has very different dynamic architectures in its ATP and ADP-bound forms, with different elements of the protein mechanically coupled together and sampling different structures.

Actin is a member of the actin/hexokinase/Hsp70 superfamily, which is characterized by a common domain consisting of two lobes with a NBS between them^54,55^. Superfamily members share little sequence similarity and perform different functions, but they have striking similarities in structure, dynamics and regulation. Hsp70 plays important roles in protein folding through adenine nucleotide-dependent cycles of binding and release of protein substrates. The molecule consists of two domains, the actin-like nucleotide binding domain (NBD) and a substrate binding domain (SBD) connected by a peptide linker. Like actin, the NBD of Hsp70 adopts nearly identical structures when crystallized in its ATP- and ADP-bound forms, yet biochemical data indicate that nucleotide controls communication between the NBD and SBD, and consequently interactions with the substrate, suggesting functional differences may arise from differences in dynamics. Consistent with this idea, residual dipolar coupling measurements on the AMPPNP and ADP forms of the NBD revealed significant rotations for different NBD SDs in response to nucleotide binding^56,57^. Similarly, comparison of the NMR chemical shifts of ATP- and ADP-bound NBD identified differences in elements corresponding to SD1, SD3 and SD4 in addition to changes clustered around the NBS^58^. Changes in the region equivalent to the hydrophobic groove of actin appear to be responsible for nucleotide-dependent binding of the interdomain linker in Hsp70 to the analogous groove, controlling communication to the SBD active site^59^. Thus, for both actin and Hsp70, nucleotide controls allosteric communication across spatially distant regions of the NBD, modulating interactions with other proteins, intermolecularly for actin and intramolecularly for Hsp70.

In the ‘nucleation and elongation’ model of actin polymerization, the energetically unfavorable formation of a dimer or trimer nucleus is followed by favorable monomer additions to produce and elongate a filament. A recent study has suggested that nucleation is unfavorable because only a small fraction of the actin monomers in solution adopt a nucleation-competent conformation that can form oligomers^14^. Our data are consistent with such a model, as we show that ATP-actin exists in solution as a rapidly equilibrating ~99:1 mixture of conformations, and that a K118N mutation that approximately doubles the population of the minor species nucleates faster than the wild-type protein. Thus, the minor species identified by NMR could be the nucleation-competent species suggested biochemically. Minimally, this species appears to be on-pathway to nucleation. Within this framework, we find that while ADP* is populated to a higher degree than ATP*, its chemical shifts, and thus its conformation, are different. This suggests that the oligomerization of ADP-actin should be even slower, and nucleation more strongly disfavored than ATP-actin, because the nucleation-competent species is even less populated.

What might the conformation of this species be? Without comparison to the chemical shifts of known structures, it is not possible to discern the conformation of weakly populated states from their methyl chemical shifts alone. Nevertheless, comparison of the structures of G- and F-actin reveals two structural changes that occur upon polymerization: the two lobes of actin rotate to flatten the molecule^10^, and the pocket above the W-loop opens to accommodate the D loop of the longitudinally adjacent subunit in the filament^11^. One or both of these changes may occur to form an oligomeric actin nucleus. It is thus possible that the equilibrium we observe in solution may represent transitions to the flattened and/or open W-loop conformations. Further studies are needed to understand the dynamics of G-actin in structural terms.

In conclusion, our data suggest a model where ATP- and ADP-bound G-actin have different ground states and also populate different high-energy states. The excited state of ATP-actin also appears to be on the pathway to nucleation, suggesting a contribution of dynamics to nucleotide-dependent assembly behavior. Finally, disease-causing mutants of actin may act by perturbing the dynamic landscape of the protein, leading to biochemical defects and consequent cellular impairment.

## Methods

### Bacterial strains

MACH1 (Invitrogen) *Escherichia coli* strain was used for passage during cloning of plasmid DNA.

### Yeast strains

The pPICZB vector was used to clone polymerization-incompetent *Drosophila* 5C actin. All recombinant proteins were expressed in the GS115 strain of *P. pastoris* cells growing in buffered minimal glycerol or methanol medium containing histidine (BMGH/BMMH) media.

### Cloning, mutagenesis and expression in *P. pastoris*

The gene encoding the polymerization-incompetent *Drosophila* 5C actin mutant (D286A, V287A, D288A) was cloned into pPICZ B (Invitrogen) using EcoRI and NotI restriction sites. The construct used to generate actin samples for NMR contained an N-terminal hexahistidine tag fused with human thymosin β4 followed by a TEV protease recognition sequence. The construct was transformed via electroporation into the GS115 strain of *P. pastoris* as described by the manufacturer (EasySelect, Invitrogen). Site-specific Ile to Val or Leu actin mutants used for Ile assignments (see below) were generated using Quik-Change site-directed mutagenesis (Stratagene). All constructs were verified by Sanger sequencing. Perdeuterated ^1^H/^13^C Ile δ1-methyl-labeled DVD G-actin samples were expressed as described by Clark et al.^37^ and Ali and others^38^.

### Purification of G-actin

DVD-actin was purified largely as described previously^37,38^. All procedures were performed at 4 °C. Cells collected from 2 l of culture were suspended in 50 ml of lysis buffer containing 50 mM Tris (pH 8.0), 10 mM imidazole (pH 7.5), 300 mM KCl, 0.2 mM ATP, 0.1 mM CaCl_2_, 1 mM PMSF, 1 mM β-mercaptoethanol (BME), leupeptin (1 μg ml^−1^), 1 mM benzamidine and antipain (1 μg ml^−1^). Resuspended cells were lysed by four passes through a microfluidizer (Microfluidics M-110P) at 25,000 psi. Cell debris was removed by centrifugation at 48,000*g* for 45 min. The resulting supernatant was loaded on 15 ml of Ni-NTA (Qiagen) slurry, pre-equilibrated with lysis buffer (without PMSF, leupeptin, benzamidine and antipain) followed by extensive washes (10 column volumes (CV), 150 ml) with buffers wash buffer 1 (WBI) (20 mM Tris pH 8, 10 mM imidazole (pH 7.5), 1 mM BME, 500 mM KCl, 0.2 mM ATP, 0.1 mM CaCl_2_) and wash buffer 2 (WBII) (20 mM Tris pH 8, 20 mM imidazole, 1 mM BME, 100 mM KCl, 0.2 mM ATP, 0.1 mM CaCl_2_). (His)_6_-thymosin β4 actin was eluted in two 75-ml fractions of WBII buffer containing 500 mM imidazole at pH 7.5. The (His)_6_-thymosin β4 tag was removed by treatment with TEV protease, overnight at 4 °C. The reaction mixture was diluted (3×) with ion exchange buffer A (10 mM imidazole, pH 7.5, 0.1 mM CaCl_2_, 0.2 mM ATP, 1 mM BME) and loaded on a source15Q anion exchange column (GE Healthcare) equilibrated with buffer A. Actin was eluted with a linear gradient of buffer B (buffer A containing 1 M NaCl). Actin-containing fractions were pooled and loaded onto 5 ml of Ni-NTA beads to remove residual TEV and (His)_6_-thymosin β4 tag. The column flowthrough was concentrated using an Amicon centricon (molecular weight cutoff (MWCO) of 10 kDa, 15 ml, Millipore) to ~15 ml and loaded on an SD200 column (GE Healthcare, 320 ml) pre-equilibrated with gel filtration (GF) buffer (10 mM HEPES, pH 7, 50 mM KCl, 0.2 mM CaCl_2_, 0.2 mM ATP, 1 mM TCEP, 1 mM NaN_3_). Actin fractions were pooled, concentrated and dialyzed into NMR buffer (10 mM HEPES pH 7, 50 mM KCl, 0.2 mM CaCl_2_, 0.2 mM ATP, 1 mM TCEP, 1 mM NaN_3_ in 100% D_2_O). This procedure results in Ca^2+^-ATP-actin.

### Preparation of Ca^2+^-ADP-actin

We initially generated Ca^2+^-ADP-actin using a modified version of a previously published protocol^60^. Purified Ca^2+^-ATP-actin in GF buffer was concentrated at 4 °C to 100 ± 10 μM (300 μl) using an Amicon centricon (MWCO 10 kDa, 5 ml, Millipore) and transferred to a 1.5-ml Eppendorf tube on ice. To this solution, 3 mM MgCl_2_, 1 mM dextrose and 40 U ml^−1^ of hexokinase (Sigma, H4502-2.5KU) were added. The reaction mixture was incubated on ice for 6–8 h. Conversion of Ca^2+^-ATP to Mg^2+^-ADP-bound G-actin was monitored by periodically warming the sample to room temperature and recording ~30-min ^1^H/^13^C HMQC spectra (Extended Data Fig. 2b). On completion of the reaction, Mg^2+^-ADP-actin was transferred into a dialysis cassette (Slide-A-Lyzer dialysis cassette G2 10,000 MWCO, Thermo Fisher Scientific, cat. no. 87729) and dialyzed against buffer (250 ml) containing 10 mM HEPES (pH 7.0), 10 mM CaCl_2_, 50 μM ADP (pre-treated with hexokinase overnight to remove contaminating ATP at 4 °C), 1 mM TCEP, 50 mM KCl, 1 mM NaN_3_ for 3 h. Finally, the resulting material was dialyzed against NMR buffer (50 ml) containing 10 mM HEPES (pH 7.0), 10 mM CaCl_2_, 50 μM ADP (pre-treated with hexokinase overnight to remove contaminating ATP at 4 °C), 1 mM TCEP, 50 mM KCl, 1 mM NaN_3_ in 100% D_2_O. Conversion of Mg^2+^-ADP- to Ca^2+^-ADP-actin was confirmed by recording ~30-min ^1^H/^13^C HMQC spectra (Extended Data Fig. 2b).

Although this method produced Ca^2+^-ADP G-actin suitable for short, simple NMR experiments, it was difficult to fully exchange Mg^2+^ for Ca^2+^, and the sample tended to precipitate and/or degrade during the relatively long process. We thus developed a more rapid and robust protocol, which is complete in ~1 h, and produces material that is stable at room temperature for ~30 h, enabling us to acquire high-quality CPMG datasets. The method is applicable to non-polymerizable DVD actin. Because yeast hexokinase has both glucose phosphorylation and ATPase activity in the presence of its optimal divalent metal ion, Mg^2+^, incubation in the presence of ATP results in production of variable amounts of phosphate ion (Pi), which contaminates Ca^2+^-ADP-actin. However, Ca^2+^ ions inhibit the ATPase activity of yeast hexokinase while retaining some ability to phosphorylate glucose^61^. Thus, to avoid the generation of Pi during conversion of ATP-actin to ADP-actin, we performed the hexokinase reaction in the presence of 0.5 mM CaCl_2_ with no MgCl_2_. Concentrated Ca^2+^-ATP-actin in GF buffer was incubated with 0.5 mM CaCl_2_, 2 mM dextrose and 40 U ml^−1^ hexokinase on ice for ~1 h. The progress of the reaction was monitored and confirmed by acquisition of 30-min ^1^H/^13^C HMQC spectra. The reaction mixture containing Ca^2+^-ADP-actin was transferred into a dialysis cassette (Slide-A-Lyzer dialysis cassette G2, 10,000 MWCO, Thermo Fisher Scientific, cat. no. 87729) and dialyzed against NMR buffer containing 10 mM HEPES (pH 7.0), 50 mM KCl, 0.5 mM CaCl_2_, 0.2 mM ADP, 1 mM TCEP, 1 mM NaN_3_ in 100% D_2_O.

### Nuclear magnetic resonance spectroscopy

All NMR data were acquired on Agilent 600- or 800-MHz spectrometers equipped with 5 mM quadruple resonance pulsed field gradient, cryogenic probes at 298 K. Samples for NMR experiments typically contained 50–220 μM actin in 10 mM HEPES (pH 7.0), 0.2/0.5 mM CaCl_2_ (ATP/ADP-bound actin sample), 0.2 mM ATP/ADP, 1 mM TCEP, 50 mM KCl and 1 mM NaN_3_ in 100% D_2_O. The sample volume was 270 μl in a D_2_O susceptibility-matched Shigemi tube.

#### Line broadening measurements

To quantify the concentration-dependent aggregation/oligomerization of DVD G-actin, we measured the linewidth at half height of the resolved peaks in 2D ^1^H/^13^C HMQC^62^ spectra recorded at 600 MHz. The spectra were acquired on freshly prepared, protonated ^13^C Ile δ1-methyl-labeled G-actin at 50, 105 and 220 μM. To ensure optimal digital resolution, data were acquired with spectral widths of 8,021.8 Hz and 1,950 Hz, affording digital resolutions of 15.6 Hz per point (63.8 ms) and 15.2 Hz per point (65.6 ms) in the ^1^H and ^13^C dimensions, respectively. An inter-scan delay of 1.2 s was employed between successive transients.

#### Measurement of side chain methyl dynamics

Side chain dynamics were measured on perdeuterated ^1^H/^13^C Ile δ-methyl-labeled samples using methyl CPMG relaxation dispersion experiments^40^. In all experiments the samples contained 100 ± 10 μM actin. Datasets were acquired at 600- and 800-MHz field strengths.

#### ^1^H/^13^C multiple-quantum Carr–Purcell–Meiboom–Gill relaxation dispersion

^1^H/^13^C MQ CPMG experiments were performed using the pulse sequence described by Korzhnev and colleagues^40^. Relaxation dispersion profiles were recorded in an interleaved fashion with a constant relaxation time of 25 ms. Different values of CPMG frequency (*ν*_cpmg_) ranging from 80 Hz to 880 Hz (at 600 MHz) or 80 Hz to 1,000 Hz (at 800 MHz) were arrayed during the constant relaxation time. Datasets included *ν*_cpmg_ duplicates to assess experimental errors. All experiments at 600 MHz were performed with acquisition times of 63.8 ms and 38.1 ms in the ^1^H and ^13^C dimensions using 16 scans per free induction decay. A recycle delay of 1.5 s was employed between successive transients, producing acquisition times of ~17 h for a complete dataset. Dispersion data at 800 MHz were recorded with 63.8-ms and 27.7-ms acquisition times in the ^1^H and ^13^C dimensions, respectively. A recycle delay of 2 s was employed between successive transients, producing acquisition times of ~20 h for a complete dataset.

#### ^1^H single-quantum transverse relaxation optimized spectroscopy Carr–Purcell–Meiboom–Gill relaxation dispersion

To distinguish contributions to relaxation from ^13^C and ^1^H methyl spins and extract accurate exchange parameters, we performed TROSY-based ^1^H SQ relaxation dispersion experiments^41^ on perdeuterated ^1^H/^13^C Ile δ1-methyl-labeled actin. Experiments were recorded in interleaved fashion and relaxation dispersion profiles were collected as pseudo 3D datasets with *ν*_cpmg_ values ranging between 66 Hz and 1,000 Hz (with constant-time CPMG element, *T*_relax_ = 30 ms) for ATP-actin or 80 Hz and 1,000 Hz (*T*_relax_ = 25 ms) for ADP-actin. Duplicate *ν*_cpmg_ data were included in all datasets for error analysis. A relaxation delay of 2 s was employed between successive transients, and the total measurement time for each dataset was ~25 h.

### Data processing

All NMR data were processed using NMRPipe/NMRDraw^63^. Directly and indirectly detected time-domain data were processed by applying a 90° phase-shifted squared sine bell or sine bell or Gaussian bell. Zero-filling was employed before Fourier transformation. Peak intensities were measured either with FuDA (http://pound.med.utoronto.ca/software) or nmrPipe or the Analysis module in CCPNMR^64^.

### Carr–Purcell–Meiboom–Gill data analysis

The data were analyzed according to protocols described by Mulder and colleagues^65^. The effective decay rate (*R*_2eff_) was calculated by the equation

$${R_{2{\rm{eff}}}} = {- {\frac{1}{{T_{\rm{relax}}}}}\,{{{\mathrm{ln}}}}{\left( {\frac{{I_{\rm{vcpmg}}}}{{I_0}}} \right)}}$$

where *I*_0_ is the peak intensity in a reference spectrum recorded without the relaxation delay (*T*_relax_)^66^. Residues showing relaxation dispersion were initially fit individually to a two-state exchange model to extract the exchange parameters *k*_ex_, populations *p*_a_, *p*_b_ (where *p*_a_ and *p*_b_ are the populations of ground and excited states, respectively) and chemical shift differences between the ground and excited states (Δ*ω*), yielding residuals, ${\chi _{\rm{indiv}}^2}$ (refs. ^42^^,^^65^). The data were then globally fit, yielding residuals ${\chi _{\rm{group}}^2}$ for each residue. Residues with ${{\chi _{\rm{group}}^2}/{\chi _{\rm{indiv}}^2}}{>} 2$ were removed from the analysis and global fitting repeated. This process was iterated until ${{\chi _{\rm{group}}^2}/{\chi _{\rm{indiv}}^2}}{<}2$ for all remaining residues. Fitting was performed using the program chemex (https://github.com/gbouvignies/chemex). Errors were estimated assuming a two-state exchange process and could be underestimated in the presence of more than two states. *k*_ex_ is the sum of *k*_AB_ and *k*_BA_, where *k*_AB_ and *k*_BA_ are the rate constants for the conversion of ground to excited state and excited to ground state, respectively, and can be expressed as

$${k_{\rm{AB}}} = {{p_{\rm{b}}}\,{k_{\rm{ex}}}}$$

$${k_{\rm{BA}}} = {{\left( {1 - {p_{\rm{b}}}} \right)}{k_{\rm{ex}}}}$$

### Δ*ω* sign determination

The sign of Δ*ω* was determined by comparing either a pair of HSQC spectra recorded at different magnetic fields or HSQC and HMQC (S/MQ) spectra obtained at a single field^67^, enabling calculation of the excited-state chemical shifts.

### Reporting Summary

Further information on research design is available in the Nature Research Reporting Summary linked to this Article.

## Online content

Any methods, additional references, Nature Research reporting summaries, source data, extended data, supplementary information, acknowledgements, peer review information; details of author contributions and competing interests; and statements of data and code availability are available at 10.1038/s41594-022-00743-5.

## Supplementary information

Supplementary Information Actin 5C amino acid sequence used in this study.

Reporting Summary

## Data Availability

The BMRB (Biological Magnetic Resonance Bank) accession number for the chemical shift assignments of Ile δ1-methyl groups in Ca^2+^-ATP-actin reported in this Article is 50918. The amino acid sequence reported in this Article is provided in the Supplementary Information. The PDB codes used in this study are 2HF3 (https://www.rcsb.org/structure/2hf3), 2HF4 (https://www.rcsb.org/structure/2hf4), 1NWK (https://www.rcsb.org/structure/1nwk), 1J6Z (https://www.rcsb.org/structure/1J6Z) and 6DJM (https://www.rcsb.org/structure/6DJM). Source data are provided with this paper.

## Source data

Source Data Extended Data Fig. 1 Uncropped gels.
