# Supplementary figures and images for: Bound nucleotide can control the dynamic architecture of monomeric actin
Source: Nat Struct Mol Biol. Author manuscript; Available in PMC 2022 Apr 20. (PMC9010300; doi:10.1038/s41594-022-00743-5)

Source Data Extended Data Figs. 1b, c, d

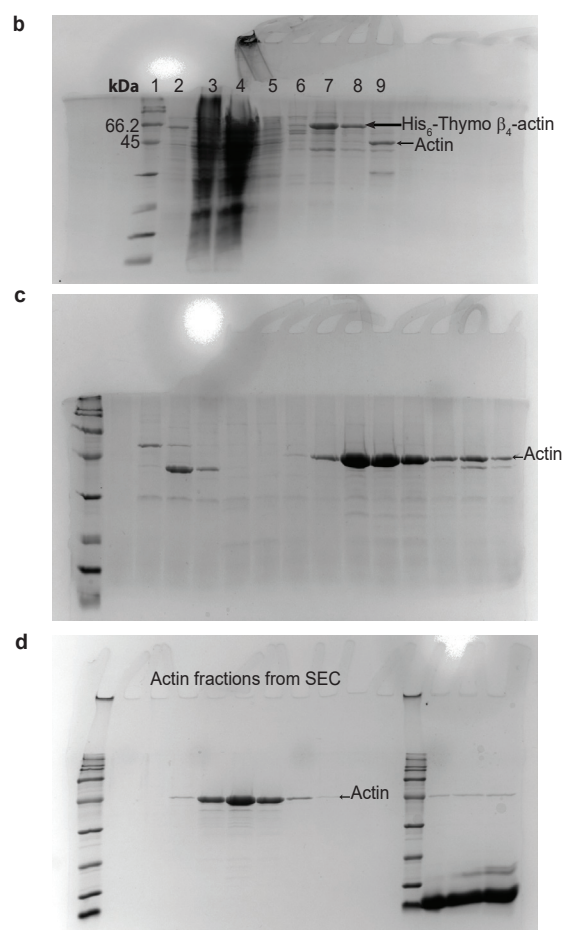

Supplement: Source data extended data fig 1 [file NIHMS1794528-supplement-Source_data_extended_data_fig_1.pdf]
