# Supplementary material for: Bound nucleotide can control the dynamic architecture of monomeric actin
Source: Nat Struct Mol Biol. Author manuscript; Available in PMC 2022 Apr 20. (PMC9010300; doi:10.1038/s41594-022-00743-5)
Supplement: Supplementary Information [file NIHMS1794528-supplement-Supplementary_Information.pdf]

Bound nucleotide can control the dynamic architecture of monomeric actin

Rustam Ali[1,3*], Jacob A. Zahm[1,2,3], Michael K. Rosen[1*]

[1]Department of Biophysics, Howard Hughes Medical Institute, UT Southwestern Medical Center, Dallas, TX, 75390, USA

[2]Present address: Department of Biological Chemistry and Molecular Pharmacology, Harvard Medical School, Boston, MA 02115, USA

[3]Equal contributions

*Corresponding authors: michael.rosen@utsouthwestern.edu (M.K.R.), rustam.ali@utsouthwestern.edu (R.A.)

Amino acid sequence number reported in this study follows the following sequencing numbering:

>3EL2_1|Chain A|Actin-5C|Drosophila melanogaster (7227)

```
CDEEVAALVV DNGSGMCKAG FAGDDAPRAV FPSIVGRPRH QGVMVGMGQK DSYVGDEAQS  60
KRGILTLKYP IEHGIVTNWD DMEKIWHHTF YNELRVAPEE HPVLLTEAPL NPKANREKMT  120
QIMFETFNTP AMYVAIQAVL SLYASGRTTG IVLDSGDGVS HTVPIYEGYA LPHAILRLDL  180
AGRDLTDYLM KILTERGYSF TTTEEREIVR DIKEKLCYVA LDFEQEMATA ASSSSLEKSY  240
ELKDGQVITI GNERFRCPEA LFQPSFLGME ACGIHETTYN SIMKCDVDIR KDLYANTVLS  300
GGTTMYPGIA DRMQKEITAL APSTMKIKII APPERKYSVW IGGSILASLS TFQQMWISKQ  360
EYDESGPSIV HRKCF 375
```
