# Supplementary material for: Bound nucleotide can control the dynamic architecture of monomeric actin
Source: Nat Struct Mol Biol. 2022 Mar 24;29(4):320–8. doi: 10.1038/s41594-022-00743-5 (PMC9010300; doi:10.1038/s41594-022-00743-5)
Supplement: Supplementary file 1 [file 41594_2022_743_MOESM1_ESM.pdf]

nature portfolio

https://doi.org/10.1038/s41594-022-00743-5

Supplementary information

Bound nucleotide can control the dynamic architecture of monomeric actin

In the format provided by the authors and unedited

Bound nucleotide can control the dynamic architecture of monomeric actin

Rustam Ali[1,3*], Jacob A. Zahm[1,2,3], Michael K. Rosen[1*]

[1]Department of Biophysics, Howard Hughes Medical Institute, UT Southwestern Medical Center, Dallas, TX, 75390, USA

[2]Present address: Department of Biological Chemistry and Molecular Pharmacology, Harvard Medical School, Boston, MA 02115, USA

[3]Equal contributions

*Corresponding authors: michael.rosen@utsouthwestern.edu (M.K.R.), rustam.ali@utsouthwestern.edu (R.A.)

Amino acid sequence number reported in this study follows the following sequencing numbering:

>3EL2_1|Chain A|Actin-5C|Drosophila melanogaster (7227)

CDEEVAALVV DNGSGMCKAG FAGDDAPRAV FPSIVGRPRH QGVMVGMGQK DSYVGDEAQS **60**

KRGILTLKYP IEHGIVTNWD DMEKIWHHTF YNELRVAPEE HPVLLTEAPL NPKANREKMT **120**

QIMFETFNTP AMYVAIQAVL SLYASGRTTG IVLDSGDGVS HTVPIYEGYA LPHAILRLDL **180**

AGRDLTDYLM KILTERGYSF TTTEEREIVR DIKEKLCYVA LDFEQEMATA ASSSSLEKSY **240**

ELKDGQVITI GNERFRCPEA LFQPSFLGME ACGIHETTYN SIMKCDVDIR KDLYANTVLS **300**

GGTTMYPGIA DRMQKEITAL APSTMKIKII APPERKYSVW IGGSILASLS TFQQMWISKQ **360**

EYDESGPSIV HRKCF **375**
