# Supplementary material for: Bound nucleotide can control the dynamic architecture of monomeric actin
Source: Nat Struct Mol Biol. 2022 Mar 24;29(4):320–8. doi: 10.1038/s41594-022-00743-5 (PMC9010300; doi:10.1038/s41594-022-00743-5)
Supplement: Supplementary file 2 [file 41594_2022_743_MOESM2_ESM.pdf]

nature portfolio

Corresponding author(s): Rustam Ali; Michae K. Rosen

Last updated by author(s): Jun 21, 2021

Reporting Summary

Nature Portfolio wishes to improve the reproducibility of the work that we publish. This form provides structure for consistency and transparency in reporting. For further information on Nature Portfolio policies, see our Editorial Policies and the Editorial Policy Checklist.

Statistics

For all statistical analyses, confirm that the following items are present in the figure legend, table legend, main text, or Methods section.

n/a	Confirmed	
☐	☒	The exact sample size (*n*) for each experimental group/condition, given as a discrete number and unit of measurement
☐	☒	A statement on whether measurements were taken from distinct samples or whether the same sample was measured repeatedly
☒	☐	The statistical test(s) used AND whether they are one- or two-sided<br>*Only common tests should be described solely by name; describe more complex techniques in the Methods section.*
☒	☐	A description of all covariates tested
☒	☐	A description of any assumptions or corrections, such as tests of normality and adjustment for multiple comparisons
☐	☒	A full description of the statistical parameters including central tendency (e.g. means) or other basic estimates (e.g. regression coefficient) AND variation (e.g. standard deviation) or associated estimates of uncertainty (e.g. confidence intervals)
☒	☐	For null hypothesis testing, the test statistic (e.g. *F*, *t*, *r*) with confidence intervals, effect sizes, degrees of freedom and *P* value noted<br>*Give P values as exact values whenever suitable.*
☒	☐	For Bayesian analysis, information on the choice of priors and Markov chain Monte Carlo settings
☒	☐	For hierarchical and complex designs, identification of the appropriate level for tests and full reporting of outcomes
☒	☐	Estimates of effect sizes (e.g. Cohen's *d*, Pearson's *r*), indicating how they were calculated

Our web collection on statistics for biologists contains articles on many of the points above.

Software and code

Policy information about availability of computer code

Data collection	Agilent varian 600/800 MHz, VNMRJ 4.2
Data analysis	NMRPipe (https://www.ibbr.umd.edu/nmrpipe/install.html), CCPNMR (Analysis 2.4.2) (https://www.ccpn.ac.uk/v2-software/downloads/stable), ChemEx (https://github.com/gbouvignies/ChemEx), pymol (https://pymol.org/2/), ligplot+ (https://www.ebi.ac.uk/thornton-srv/software/LigPlus/), chimera 1.16 (https://www.cgl.ucsf.edu/chimera/), FuDa (https://www.ucl.ac.uk/hansen-lab/fuda/)

For manuscripts utilizing custom algorithms or software that are central to the research but not yet described in published literature, software must be made available to editors and reviewers. We strongly encourage code deposition in a community repository (e.g. GitHub). See the Nature Portfolio guidelines for submitting code & software for further information.

Data

Policy information about availability of data

All manuscripts must include a data availability statement. This statement should provide the following information, where applicable:

- Accession codes, unique identifiers, or web links for publicly available datasets
- A description of any restrictions on data availability
- For clinical datasets or third party data, please ensure that the statement adheres to our policy

Chemical shifts for assigned 1H/13C delta-1 methyl groups of Ile have been deposited in BMRB with accession code 50918. Current status: Release on publication. Plasmids used in this study are available upon request. This study used protein structures with PDB IDs: 2HF3 (https://www.rcsb.org/structure/2hf3), 2HF4 (https://www.rcsb.org/structure/2hf4), 1NWK (https://www.rcsb.org/structure/1nwk), 1J6Z (https://www.rcsb.org/structure/1J6Z), 6DJM (https://www.rcsb.org/structure/6DJM).

March 2021

Field-specific reporting

Please select the one below that is the best fit for your research. If you are not sure, read the appropriate sections before making your selection.

☒ Life sciences ☐ Behavioural & social sciences ☐ Ecological, evolutionary & environmental sciences

For a reference copy of the document with all sections, see nature.com/documents/nr-reporting-summary-flat.pdf

Life sciences study design

All studies must disclose on these points even when the disclosure is negative.

Sample size	The number of time domain data points sampled in NMR measurements was decided to provide resolutions of 36.1 Hz/pt to 15.2 Hz/pt in the 13C dimension, and 15.6 Hz/pt in the 1H dimension, so that measured linewidths are accurate and not limited by digital resolution.
Data exclusions	No experimental data were excluded.
Replication	The error bars in R2eff represent the SD (standard deviation), calculated from the two and three replicated data points for 600 and 800 MHz data, respectively.
Randomization	All samples were analyzed and reported individually; there was no allocation into groups.
Blinding	Blinding was not necessary because there are not subjective assessments in NMR data analyses.

Reporting for specific materials, systems and methods

We require information from authors about some types of materials, experimental systems and methods used in many studies. Here, indicate whether each material, system or method listed is relevant to your study. If you are not sure if a list item applies to your research, read the appropriate section before selecting a response.

Materials & experimental systems

n/a	Involved in the study	
☒	☐	Antibodies
☒	☐	Eukaryotic cell lines
☒	☐	Palaeontology and archaeology
☒	☐	Animals and other organisms
☒	☐	Human research participants
☒	☐	Clinical data
☒	☐	Dual use research of concern

Methods

n/a	Involved in the study	
☒	☐	ChIP-seq
☒	☐	Flow cytometry
☒	☐	MRI-based neuroimaging
